# High capacity DNA data storage with variable-length Oligonucleotides using repeat accumulate code and hybrid mapping

**DOI:** 10.1186/s13036-019-0211-2

**Published:** 2019-11-21

**Authors:** Yixin Wang, Md Noor-A-Rahim, Jingyun Zhang, Erry Gunawan, Yong Liang Guan, Chueh Loo Poh

**Affiliations:** 1 School of Electrical & Electronic Engineering, Nanyang Technological University, Singapore, 639798 Singapore; 2 Department of Biomedical Engineering, National University of Singapore, Singapore, 117583 Singapore; 3 NUS Synthetic Biology for Clinical and Technological Innovation (SynCTI), Centre for Life Sciences, National University of Singapore, Singapore, 117456 Singapore; 4 School of Computer Science and IT, University College Cork, College Road, Cork, T12 K8AF Ireland

**Keywords:** DNA data storage, Long term data storage, Next-generation information storage

## Abstract

**Background:**

With the inherent high density and durable preservation, DNA has been recently recognized as a distinguished medium to store enormous data over millennia. To overcome the limitations existing in a recently reported high-capacity DNA data storage while achieving a competitive information capacity, we are inspired to explore a new coding system that facilitates the practical implementation of DNA data storage with high capacity.

**Result:**

In this work, we devised and implemented a DNA data storage scheme with variable-length oligonucleotides (oligos), where a hybrid DNA mapping scheme that converts digital data to DNA records is introduced. The encoded DNA oligos stores 1.98 bits per nucleotide (bits/nt) on average (approaching the upper bound of 2 bits/nt), while conforming to the biochemical constraints. Beyond that, an oligo-level repeat-accumulate coding scheme is employed for addressing data loss and corruption in the biochemical processes. With a wet-lab experiment, an error-free retrieval of 379.1 KB data with a minimum coverage of 10x is achieved, validating the error resilience of the proposed coding scheme. Along with that, the theoretical analysis shows that the proposed scheme exhibits a net information density (user bits per nucleotide) of 1.67 bits/nt while achieving 91% of the information capacity.

**Conclusion:**

To advance towards practical implementations of DNA storage, we proposed and tested a DNA data storage system enabling high potential mapping (bits to nucleotide conversion) scheme and low redundancy but highly efficient error correction code design. The advancement reported would move us closer to achieving a practical high-capacity DNA data storage system.

## Background

It has been predicted that the amount of data around the world will rise to 44 zettabytes by 2020 with 2.5 exabytes of daily data production [1]. With this ever-increasing information in the digital world, the effective way to store enormous data with high reliability, capacity and durability has been much discussed. Traditional digital storage systems (e.g., CD, DVD, flash drivers, etc.) could provide a density of around 201 GB/in^2^, but require a large physical space to store data with magnitude of zettabytes [2]. Another desirable characteristic of data storage is the long preservation duration. However, tapes, disks and other traditional mediums are only capable of storing data in tens of years due to high tendency to decay [3]. Deoxyribonucleic acid (DNA) has recently attracted much attention as its inherent features, such as high physical density and long durability, significantly accommodate the requirements of large-sized long-term storage [4].

In 2012, Church et al. stored 0.65MB of data in synthetic DNA with a physical density of 1.28PB/gram [5]. However, only partial data was retrieved due to the absence of error control code. In the following year, Goldman et al. encoded and fully recovered 0.75MB of data from synthesized DNA developed using a coding scheme that consists of data compression and error correction [6]. Various techniques have been introduced to DNA-based data storage [7–14], promoting the reality of storing extremely large amounts of digital data in a molecular drop.

**Fig. 1:**
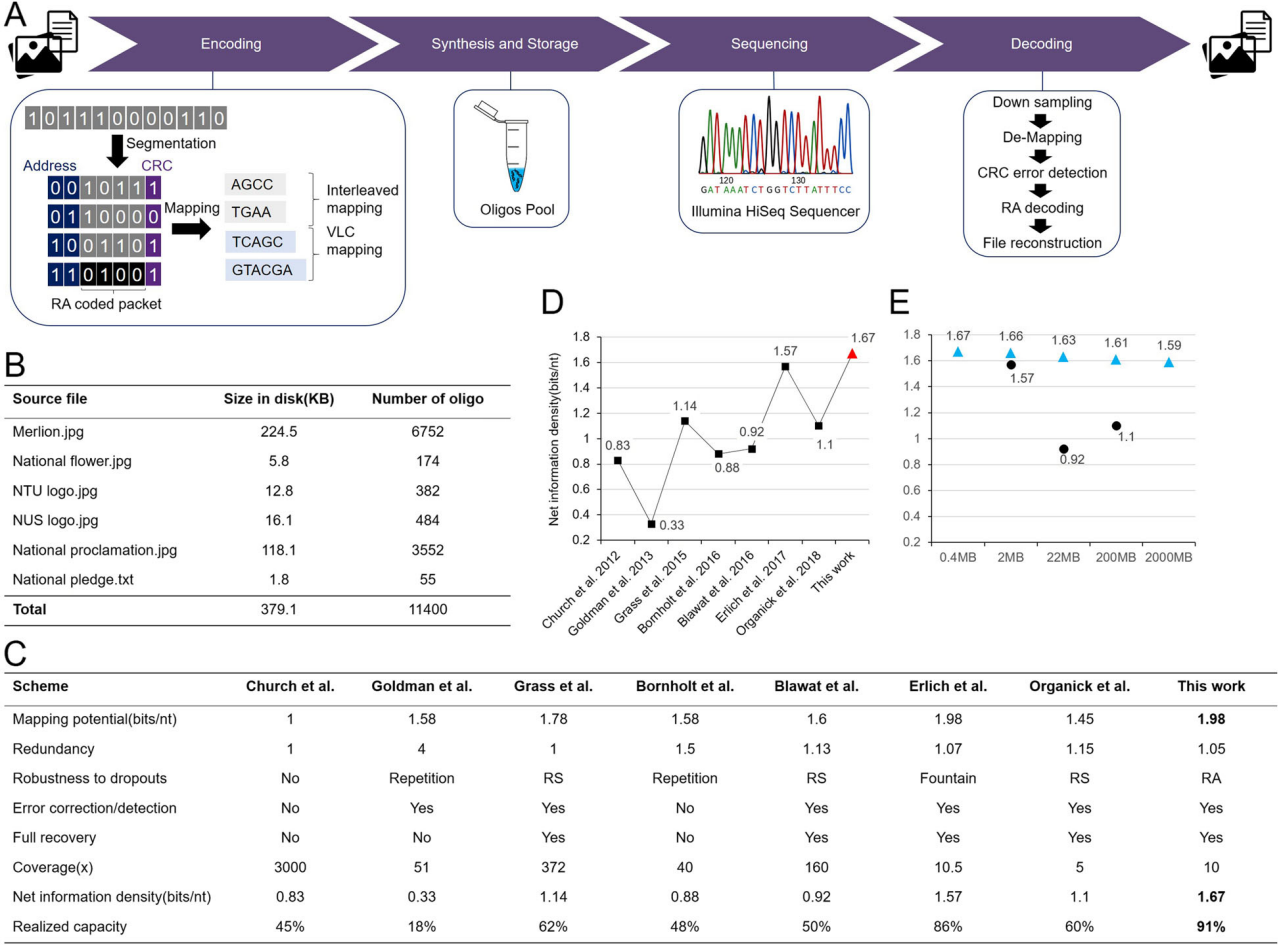
The proposed architecture of DNA data storage, stored data and results of scheme performance. (**A**) A long binary stream is segmented into source packets with the same length before performing packet level repeat accumulate encoding where parity packets are generated. All packets are attached with unique addresses to locate their orders and Cyclic Redundancy Check (CRC) to detect errors occurring interiorly. After that, the digital information is converted to DNA sequences according to the hybrid mapping strategy. The mapped DNA sequences are synthesized for storage. Then, DNA sequencing is performed to read the oligos. At the last stage, we recover the original data by performing de-mapping and decoding with the sequencing result. (**B**) Six files of total size of 379.1 KB are encoded. (**C**) By taking the comparison table in [13] as a reference, we compare existing oligo-based DNA data storage schemes where improvements are shown in several aspects of performance. (**D**) The proposed scheme achieves 1.67 bits/nt net information density, which is higher than the existing oligo-based DNA data storage schemes. (**E**) The net information density of the proposed coding scheme with different magnitudes of data size is computational analysed, in which the density slightly decreases with the exponential increase of the data size (blue triangle). The estimated density results are higher than the existing work [11, 13, 14] with the equivalent data scales (black circle)

Most previous works have dealt with two major issues faced in DNA data storage. One is to design appropriate error correction coding schemes to address the errors induced from synthesis, storage, sample preparation, and sequencing processes. Several classic error correction codes have been borrowed while with limited performance in terms of code rate, encoding/decoding complexity, and error resilience [6, 7, 11, 12, 14]. The second is to design DNA mapping strategy that converts binary data into DNA sequences subject to the biochemical constraints (i.e., balanced GC content and maximum homopolymer run limit). Different mapping strategies have been devised [5–12, 14]. However, most of them sacrificed much mapping potential (the number of bits encoded per nucleotide) to comply with the biochemical constraints, consequently lowering the net information density (the number of user bits encoded per nucleotide) (Fig. 1(C)). Together with the indexing demand for indicating the order of each stochastic stored oligo in the whole archive, the redundancies introduced by the error control and constrained mapping upper bound the amount of information data that could be reliably stored in each nucleotide, i.e., Shannon information capacity of DNA data storage [13]. Two recent works introduced additional degenerate base characters to the basic four bases, increasing the information capacity over the upper boundary (2 bits/nt) of the four-base system [15, 16]. However, compared with DNA storage with four basic bases, i.e., A/T/C/G, DNA storage with degenerate bases necessitate higher sequencing depths and improved synthesis techniques for practical usage.

In the context of DNA storage with four basic bases, a recent work of [13] achieved a significant increment in the net information density, i.e., 1.57 bits/nt, which prominently profits from their near-optimal mapping potential, i.e., stored 1.98 bits per nucleotide (bits/nt). The authors in [13] directly mapped two bits to one nucleotide before a screening post-processing. The feasibility of this instinctive mapping leverages from the harnessed fountain code [17, 18], in which limitless encoded sequences can be generated from the source data, and among them, only the valid sequences (i.e., DNA sequences that satisfy the biochemical constraints) are selected and stored in the storage. In other words, DNA fountain saved the redundancy for constrained mapping by taking advantage of the rateless feature of the fountain code. Although DNA fountain has achieved full recovery with a very high information density, the potential decoding failure and redundant addresses (which essentially decrease the net information density) arising in the fountain code, might potentially impede its practical use in DNA data storage with scalability.

To overcome the limitations of DNA fountain while achieving the competitive net information density, we are inspired to explore another practical error control code and efficient DNA mapping strategy. We thus devise a DNA data storage coding scheme using repeat-accumulate (RA) code [19] for reliable error control and a hybrid DNA mapping strategy for complying with biochemical constraints while attaining high mapping potential. In particular, a hybrid mapping scheme (consisting of two mapping methods: interleaved mapping and variable-length constrained (VLC) mapping) is developed, exhibiting close to the optimum mapping potential (i.e., 2 bits/nt) while satisfying the balanced GC content and maximum homopolymer run limit. Due to the characteristic of the hybrid mapping scheme (especially the VLC mapping), the implemented DNA data storage stores a range of variable-length oligos in an oligo pool, which proposes a different but effective format from the existing DNA data storage prototypes [5–7, 11–14]. Furthermore, a packet-level RA code is elaborately designed for DNA storage channel, offering low redundancy, low encoding/decoding complexity and high error resilience to the erroneous oligos (i.e., oligos that are subjected to errors, including insertions, deletions and substitutions during the processes of storage). Taken together with the highly efficient hybrid mapping strategy and RA code, the proposed DNA storage scheme shows robustness over biochemical mutations (full recovery from minimum coverage of 10x) and a very high net information density (1.67 bits/nt), offering a practical alternative to DNA fountain.

## Result

### A practical DNA data storage system with high capacity

We started with constructing an architecture of storing data and retrieving data from a DNA-based storage (Fig. 1(A)). The user data were first segmented into 11,400 binary user packets with each packet length of 266 bits. To correct errors occurring from any stage in the DNA storage processes including synthesis, amplification, storing, and sample preparation for sequencing, we applied a RA encoding on binary user packets where 5% redundant/parity packets were generated. With each of the 12,000 binary packets, 14 bits were added for indexing to order the stochastic oligos and 20 bits were added for Cyclic Redundancy Check (CRC) to detect the interior errors in each packet. As a result, the total number of bits associated with each packet became 300 bits (See Additional file 1: Figure S4). Afterwards, we mapped all binary sequences into DNA sequences through the proposed hybrid mapping scheme. Then the DNA sequences were sent to Twist Bioscience for oligos synthesis. After receiving the synthesized oligos pool, we amplified it using Polymerase Chain Reaction (PCR) before sending the samples to NovogeneAIT for sequencing using Illumina HiSeq. In the last stage, we analysed and decoded the sequencing data to convert the DNA records back to digital binary data. We first down-sampled the millions sequence reads from the sequencing result and performed the reverse of RA coding and mapping to reconstruct the original user data without errors, validating the feasibility of our method.

In addition to the full recovery of data using the sequencing results, we also quantitatively analysed the proposed DNA-based storage scheme and compared it with other state-of-the-art schemes, by referencing a previous comparison table [13] (Fig. 1(C)). The detailed definition of performance metrics in the table is described in Additional file 1: Section S7. In the table, we only compared with the schemes that were designed and tested with the premise of the oligo pool storage format where the single-stranded short oligos of length around 200nt were synthesized. Note that with the equivalent assumption of storing much longer DNA strands like [8], i.e., 1000bp, the proposed coding scheme remains feasible, and the net information density will increase with the length, achieving higher density than [8], i.e., 1.84 bits/base over 1.74 bits/base (see Additional file 1: Section S3).

The high net information density of 1.67 bits/nt achieved by the proposed DNA-based storage scheme (Fig. 1(D)) is mainly due to the following two techniques that we have used. Firstly, the proposed hybrid mapping scheme exhibits 1.98 bits/nt mapping potential with a small gap of 1% from the theoretical upper boundary of 2 bits/nt. Secondly, the optimized RA code for error control has a small redundancy of 1.05. Together with the 14 bits indexing and 20 bits CRC, the scheme obtains 1.67 bits/nt net information density, yielding 91% of the Shannon capacity (1.83 bits/nt with 0.5% dropout rate [13]), which is 6% more than the last highest one reported in [13] (Additional file 1: Section S3). Theoretically, compared with [13], the increase in our information density is the combined result of the slightly longer variable-length DNA oligos (151nt-159nt versus 152nt, excluding primer binding sites), the less error control redundancy (1.05 versus 1.07), and the shorter indexing (14bits versus 32bits). The length of DNA oligos are elaborately designed to make full use of the current widely available DNA synthesis techniques (TWIST Bioscience, US), which can efficiently synthesized 200nt long oligos. The optimized RA code design gives slightly reduced error control redundancy with the equivalent assumption of addressing 1.3% practical dropout rate as [13], while the full recovery with 10x coverage (10.5x in [13]) indicates that the error resilience is maintained. The most distinct difference arises in the indexing, in which we use 14 bits solely for indicating the order of encoded 12000 oligos, while [13] uses 32 bits to represent the seeds required for Luby transform which sets the basis of fountain code, resulting in redundant indexing bits.

To further verify that the high capacity performance of the proposed coding scheme maintains well with increasing data size (scalability), we estimated the net information density for encoding data size with higher magnitudes in silico, i.e., from 2MB to 2000MB. The estimated densities decrease slightly with the exponential increases of data size due to the increment of indexing length required for recording larger data size (Additional file 1: Section S3 and Fig. 1(E)). A density of 1.66 bits/nt is obtained for storing 2MB of source data, which is still 6% higher than [13]. In addition, both the RA code and hybrid mapping strategy consisting of the proposed coding scheme have a low complexity that are efficient to implement in practice. In particular, the use of RA code prevents the potential decoding failure (due to the loss of initial entries for starting decoding in the screening process) and address redundancy that may arise in DNA fountain, and the hybrid mapping achieves a very high mapping potential that is competitive with DNA fountain while avoiding high complexity that exhibits in the conventional constrained block codes.

Additionally, we computationally estimated the physical density that the proposed scheme could exhibit. Through dilution experiments, authors in [13] observed 4% dropout rate with a sample of 10pg DNA storage, which nearly approached their decoder limit (that was predetermined by the code redundancy). The RA code used in our scheme was optimally designed with a level of redundancy under the same assumption of dropout rate considered in [13]. We have also shown that theoretically our code can tolerate upto 4.75% dropout rate (Additional file 1: Figure S4), which is above the 4% dropout rate observed in sequencing 10pg sample. With similar decoding limit, our proposed scheme would likely work the same as DNA fountain in the low molecular experiments (e.g., with 10pg sample) due to the use of the same experiment pipelines, protocols, and standards. In other words, the code design at the initial stage enables that the proposed system could recover data from error-prone conditions in the dilution experiments similar to DNA fountain. Under the assumption of ∼1300 molecules per oligo in average, sequencing depth of 511x, and equivalent pipelines, protocols, and standards as the 10pg dilution experiment in DNA fountain, we could computationally estimate that our scheme will achieve a physical density of 239 PB/g $\left (\frac {266*11400/8\text {byte}}{1300*11400*1.0688*10^{-19}\text {gram}}\right)$. However, a rigorous experiment is required to verify this computationally estimated physical density.

### RA code design and hybrid mapping scheme for DNA storage

We designed an encoding method which comprises oligo-level repeat accumulate (RA) code and an efficient hybrid mapping scheme.

#### RA code design

**Fig. 2:**
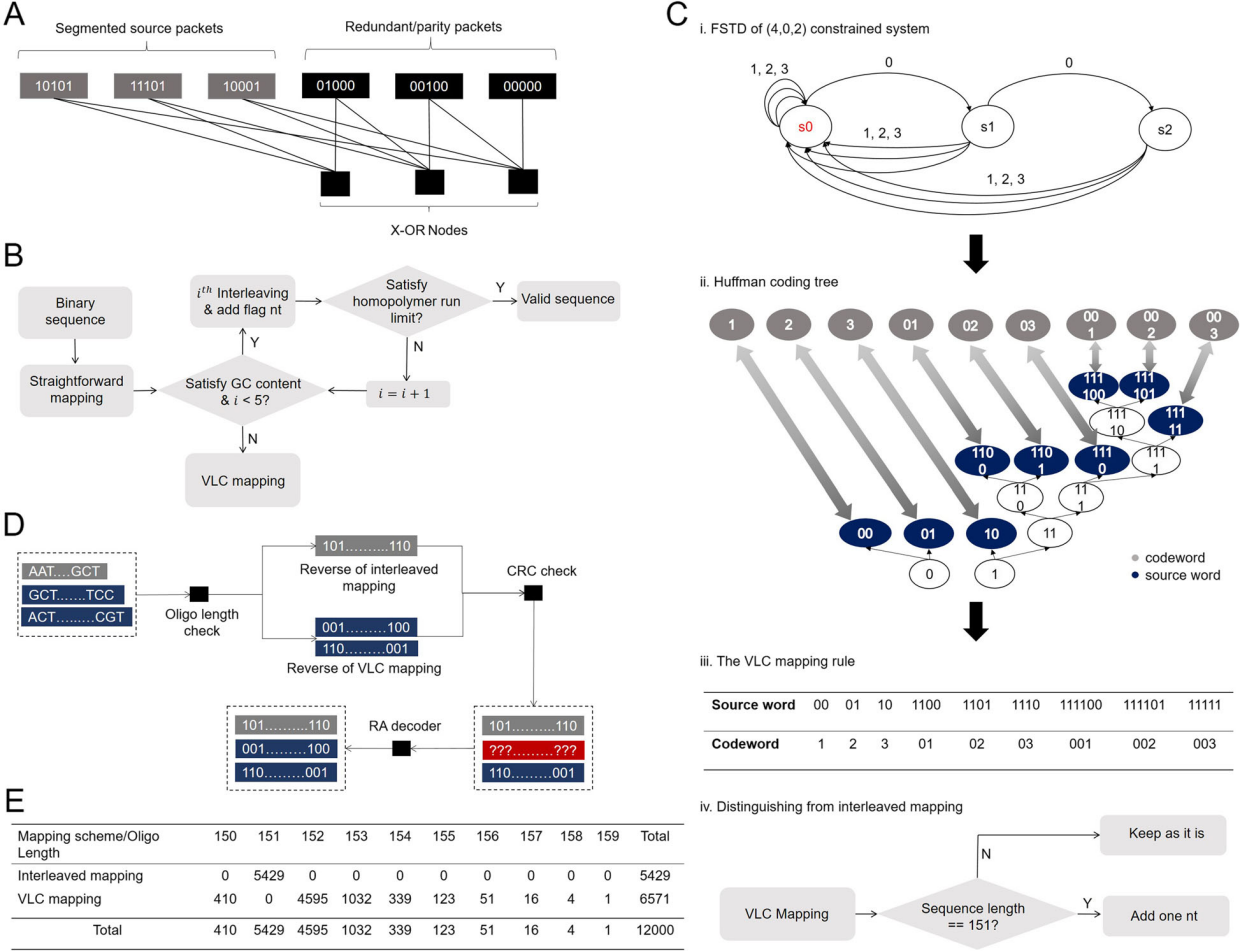
The illustration of Repeat Accumulate (RA) coding strategies and the hybrid mapping. (**A**) An example of rate $\frac {1}{2}$ packet level RA code with 3 source packets. A *i*^*th*^ parity packet at position i is generated by bit-wise modulo-2 sum of the (*i*−1)^*th*^ parity packet and the source packets that are connected to the *i*^*th*^ X-OR node. (**B**) The flow chart of the hybrid mapping. Each binary sequence is initially mapped via binary-to-quaternary mapping. With one of interleaving patterns, the interleaved sequence with the flag nucleotide appending at the end might pass the screening test where GC content and homopolymer are checked, outputting a valid sequence. Otherwise, the original binary sequence will be sent to the variable-length constrained (VLC) mapping. (**C**. i) The FSTD of a (4, 0, 2) constrained DNA storage system, where 0, 1, 2, and 3 represent four transition symbols that indicate the transitions among four nucleotide alphabets, and s0, s1 and s2 represent three different states that record the length of consecutive 0’s (no transition) in the output (4, 0, 2) constrained sequences. (**C**. ii) The generation of a Huffman coding tree. The Huffman coding tree optimizes the code rate by aligning the source word with high occurrence possibility to the codeword with short length and verse vice. (**C**. iii) The VLC mapping rule. The alignment of Huffman coding tree generates a look-up table between variable-length source words and variable-length transition codewords. (**C**. iv) The strategy for enabling the decoder to distinguish two mappings via the length of received DNA sequence. (**D**) The flow chart of the decoder. The decoder first distinguishes the mapping method the received sequence has used and performs the associative reverse. The CRC check then decides on whether the reversed binary sequence is in errors or not. Afterwards, the RA decoder works to recover all sequences in errors. (**E**) The distribution of lengths of mapped DNA sequences. The length of resultant DNA sequences ranges from 150nt to 159nt, where the interleaved mapping only generates sequences with the length of 151nt while sequences with other lengths are all generated by the VLC mapping

In traditional communication systems, RA code is used at bit-level, where redundant bits are generated to mitigate substitution errors. However, DNA storage is prone to not only substitution errors but also to insertion and deletion errors. Hence, instead of the conventional bit-level RA encoding, we designed a packet level RA encoding for DNA storage such that a packet subjected to insertion, deletion, or substitution errors could be recovered through RA decoder. As described earlier, we have segmented a large digital file into smaller packets of the same size. These packets were considered as the source packets which were used to generate the redundant or parity packets using systematic RA code Fig. 2(A). Note that every packet was incorporated with CRC to detect errors in the packet. For the packets that passed the CRC test in the decoder, we considered them as correctly recovered, while the others were regarded as dropped or erased. Thus, the overall code design problem for the DNA storage became the code design for the erasure channel. To ensure high reliability, the code design was performed by considering a slightly higher dropout probability than the actual dropout probability. In this work, we considered the actual dropout rate as 1.3% which was reported in the fountain paper [13]. Thus, we designed the RA code such that the resultant code exhibited an asymptotic threshold higher than the dropout probability of 0.013. Following the optimization procedure (see Additional file 1: Section S2), we designed a RA code of rate 0.95, which gives an asymptotic threshold of 0.0475. The resultant code shows only a gap of 0.0025 from the Shannon’s capacity limit (0.05). The simulated error correction performance of the designed RA code is shown in Additional file 1: Figure S4. Due to the rate 0.95 RA code, we generated 600 redundant/parity packets based on 11,400 source packets, receiving 12,000 binary packets in total after encoding.

#### Hybrid mapping scheme

Next, we consider representing the digital data in DNA context which we denote as DNA mapping. A DNA mapping strategy should enable the mapped oligo sequences satisfying the biochemical constraints, thus bringing stability to the storage. There are two such constraints in DNA data as following: (i) The GC content (the ratio of the total number of ’G’ and ’C’ against the total number of nucleotides in a sequence) needs to be close to 50% (ii) All homopolymer run lengths (the length of repetitively consecutive nucleotides) should be less than 4 [13]. Note that the binary-to-quaternary mapping, i.e. mapping two bits to one nucleotide, which exhibits the optimal mapping potential (2 bits/nt), does not always meet the above mentioned requirements. Instead, it often fails to comply with the maximum homopolymer run constraint. The constraints existing in DNA data storage reduce the effective mapping potential, adversely affecting the capacity of DNA data storage. Therefore, we explored the approach of designing constrained code with high code rate and developed a hybrid mapping strategy to ensure oligo sequences meet the biochemical demands with minimal sacrifice of the mapping potential.

This mapping scheme consists of two different mapping methods, namely the interleaved mapping and the VLC mapping. The first one works as the primary mapping due to its approximately optimum mapping potential, i.e. 1.995 bits/nt and the latter one works as the backup that comes into play when the first mapping fails to produce valid DNA sequences (i.e., sequences that satisfy the GC content and homopolymer run constraints). In the later mapping method, an auxiliary look-up table is constructed with low encoding and decoding complexity. Meanwhile, this method exhibits a 1.976 bits/nt mapping potential which is much higher than the block codes [20] with the equivalent complexity. The combination of these two mapping strategies results in an average mapping potential around 1.98 bits/nt with the stochastic data. In other words, in the worst-case scenario where all data is coded using VLC, we still achieved a high mapping potential estimate (1.976 bits/nt). However, in the best case when all data are mapped using the interleaved mapping, we could achieve a very high potential of 1.995 bits/nt.

The digital data first go through the interleaved mapping method to generate the DNA sequences. In the interleaved mapping method, the binary sequences are first mapped using binary-to-quaternary mapping. With the increasing oligo length, GC content constraint is often satisfied due to the stochastic feature of binary data. However, this mapping tends to fail to satisfy the homopolymer run constraint. To solve this issue, we introduce an interleaver after the binary-to-quaternary mapping, which scrambles the original order of the nucleotide sequences. After interleaving, a screening test is performed to check the homopolymer run of the resultant sequence. If the resultant sequence passes the test, that sequence is regarded as a valid sequence for synthesis, otherwise the interleaving is performed again on the original sequence with a different interleaving pattern. In this work, we consider 4 predefined interleaving patterns, where a flag nucleotide (A/T/G/C) is appended at the end of the interleaved DNA sequence to indicate the interleaving pattern (Additional file 1: Section S8). Note that the appended flag nucleotide is included in determining the homopolymer run of the sequence during the screening test. We only use one extra (flag) nucleotide to maintain high net information density. Consequently, the number of interleaving trials is limited to 4. If the sequence still fails to meet the demand after the maximum number of trials, the sequence is sent to the VLC mapping method (Fig. 2(B) and Additional file 1: Section S4).

The VLC mapping is inspired by the construction of variable-length constrained sequence (VLCS) code, commonly used to encode data into constraint-satisfying codes in constrained systems, like optical recording systems where run-length limit and DC-free issues arise [21, 22]. In DNA storage scenario where similar constraints exist, VLCS code can be effectively modified to a mapping method. Note that as we use the packet-level RA code for error control, the error propagation led by VLCS code is limited in one packet and has no influence on the overall dropout rate of the encoded sequences.

We generated this mapping rule in the following four stages. First, considering the constraint of the maximum homopolymer runs, the DNA-based storage were seen as a constrained system with run-length limit (RLL)[23], denoted by (*M*,*d*,*k*), where *M*=4,*d*=0 and *k*=2 (Additional file 1: Section S5). Accordingly, the finite state transition diagram (FSTD) of the (4,0,2) homopolymer-constrained DNA data storage was generated (Additional file 1: Section S5 and Fig. 2(C, i)). In the second stage, based on the generated FSTD, we deduced that the capacity of the (4, 0, 2) homopolymer-constrained DNA storage is 1.982 bits/nt (Additional file 1: Section S5). We also established a complete minimal set (a finite set of words whose concatenations include all possible constraint-satisfying sequences [24]), where we enumerated all the words that originate from and end in the state s0 in Fig. 2(C, i). As a result. we obtained a minimal set {1,2,3,01,02,03,001,002,003}, in which all elements are constraint-satisfying and prefix-free. These two properties ensure that any concatenation of the elements of this set produces constraint-satisfying sequences that are potential transition codewords for the constrained system. Note that the resultant transition codeword set relates to the depth and width of the concatenation. For reducing the coding complexity, we directly used the complete minimal set as the transition codeword set.

In the third stage, we used the Huffman coding tree [25] to generate an optimal mapping from the variable-length binary source word set to the above mentioned transition codeword set (Fig. 2(C, ii)). This optimal one-to-one assignment gave an average code rate of 1.976 bits/nt (Fig. 2(C, iii) and see Additional file 1: Section S5). Meanwhile, the efficiency of this mapping approaches $\sigma =\frac {1.976}{1.982}=99.7\%$, presenting only 0.3*%* gap from the capacity of the (4,0,2) constrained system. In terms of mapping potential, this mapping outperforms the block constrained code proposed in [20], in which a (4,0,2) constrained code was constructed using 39nt DNA blocks as the codewords, achieving 1.95 bits/nt mapping potential. Besides, the 39nt block code is also impractical for traditional DNA data storage where a much longer DNA sequences (codewords), i.e., 200nt, are considered. In contrast, the variable-length mapping approach has low coding complexity regardless of the overall length of the resultant oligo sequences.

In the last stage, after mapping the source words to the transition codewords in succession against each binary sequence, we performed precoding on the encoded quaternary sequences according to the change-of-state function *y*_*j*_=*y*_*j*−1_+*x*_*j*_(*mod* *M*), where *y*_*j*_ is the current output precoding symbol, *y*_*j*−1_ is the last output pre-coded symbol, *x*_*j*_ is the current input symbol, *M* is the alphabet size of the system. This precoding will transfer the encoded (*M*,*d*,*k*) constrained code to the (*M*,*d*+1,*k*+1) RLL code. We then converted the quaternary symbols from {0,1,2,3} to {’A’, ’T’, ’C’, ’G’ } and obtained the final oligo sequences that satisfying the constraint of no homopolymer runs larger than 3nt. An example of this mapping strategy can be found in Additional file 1: Section S6.

Through the hybrid mapping scheme, we generated 12,000 DNA sequences with a length distribution ranging from 150nt to 159nt (excluding 40nt of primer sites) for the binary data stream (Fig. 2(E)). Specifically, the length of sequences that mapped via the interleaved mapping became 151nt, while the length of sequences that mapped via VLC mapping ranged from 150, 152 to 159nt. Note that there was no sequence with length of 151nt that originated from VLC mapping as one nucleotide was added to make these 151nt mapped sequence to be 152nt (Fig. 2(C, iv)). The added nucleotide was to distinguish between the mapping methods. This enables the use of correct de-mapping during the recovery of the stored data in the decoder.

To retrieve data, the prepared sequences from the sequencing process are sent to the decoder to recover the user data (Fig. 2(D)). The decoder first distinguishes the mapping method. If the length of received sequence is 151nt, the decoder applies the reverse of interleaved mapping based on the flag nucleotide and the binary-to-quaternary mapping rule. Otherwise, the decoder applies the reverse of VLC mapping where the reverse of the precoding and mapping are performed. After that, each reversed binary sequence is regarded as either a correct one or an erasure one based on the CRC check. Finally, with a message passing algorithm, the RA decoder recovers all erased sequence packets based on the connections among packets.

### Sequencing results and data recovery analysis

After sequencing the synthesized oligos pool, we received over 10 million raw sequence reads in total size of 3.2 Gigabytes from NovogeneAIT. These sequences include noisy reads generated during sequencing. Based on the sequencing results firstly we analysed the reliability of the sequencing data in terms of data quality examination, A/T/G/C content distribution and error rate distribution. Based on the error analysis result, we then studied the reliability of our decoding scheme in recovering the encoded data with different sample coverages.

#### Sequencing results

**Fig. 3:**
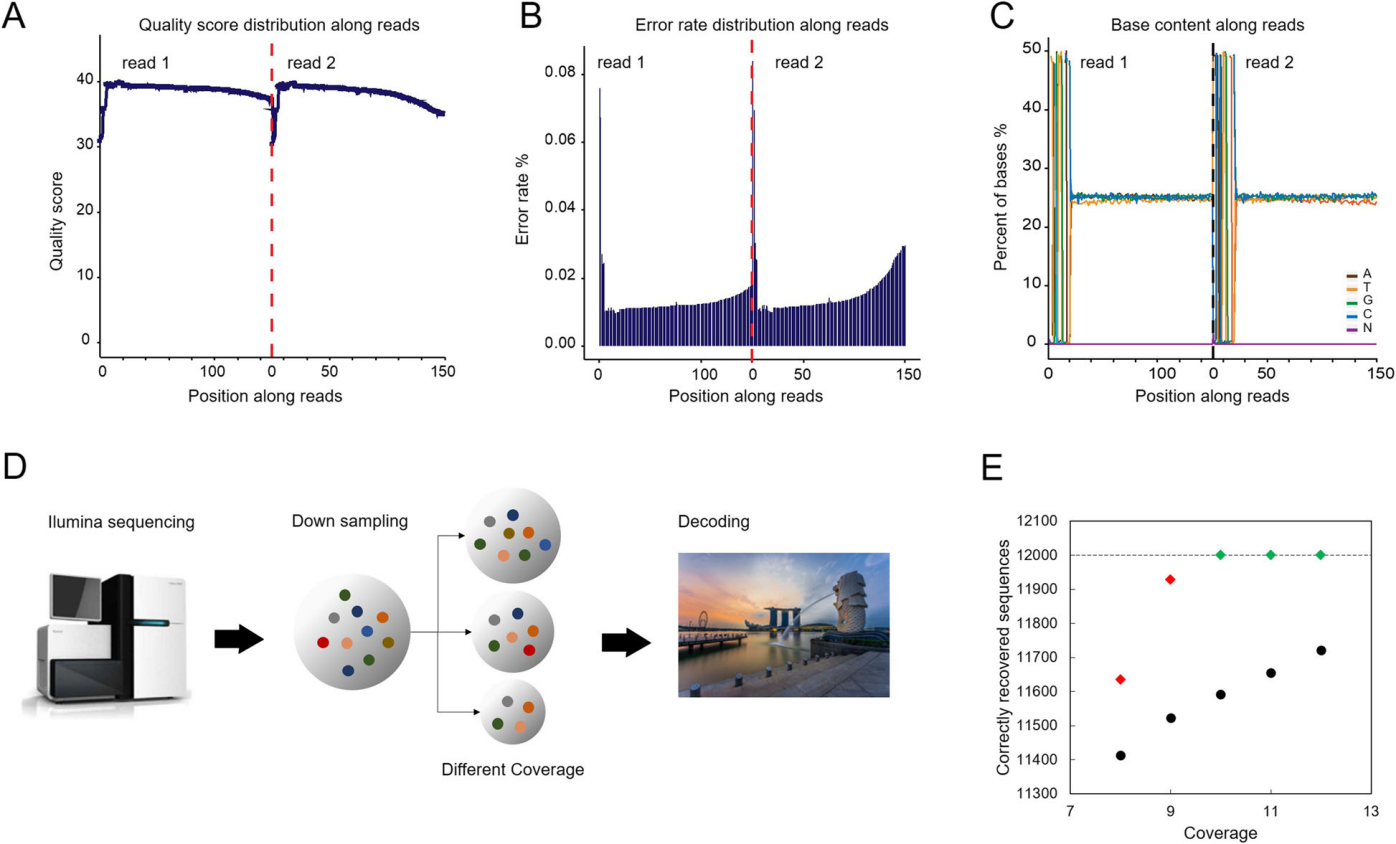
Sequencing result analysis and data recovery. (**A**) The quality value of each base position along the reads. The first half part of the x axis is for reads 1 and the latter half part is for reads 2. (**B**) The error rate of each base position along the reads. The first half part of the distribution is for reads 1 and the latter half part is for reads 2. (**C**) The base content of each base position along the reads. A/T/G/C denote the type of nucleotides and N denotes a lost nucleotide which can be any one of A/T/G/C. The distribution is separated by two reads, note that for (a), (b) and (c), read 1 and read 2 are obtained from randomly sequencing from either the end of each sequence. (**D**) The experimental procedure for data recovery. The amplified and prepared synthetic oligo samples are sequenced using Illumina HiSeq sequencing technology. With five sets of down-sampling trials, different sizes of randomly chosen portions of raw sequence reads are sent to the decoder where the stored files are recovered. (**E**) The number of correctly recovered sequences against the coverage. The black circle markers represent recovered sequences before RA decoding and diamond markers represent recovered sequences after RA decoding. Among the diamond markers, red ones represent partial recovery, while green ones represent full recovery

We analysed the quality value for each base position along the sequenced reads to evaluate the data quality. The quality score is an estimate of the reliability of the sequenced reads that relates to the error rate of each base position. It is calculated by *Q*=−10*log*_10_*e*, where *e* is the error rate of the base position [26]. The quality scores of each base of the sequencing reads range from 30 to 40 (Fig. 3(A)), representing a high quality. Further, we observe that error rate increases with the extension of sequenced reads while with an average rate of 0.015% in each base along the reads (Fig. 3(B)). This is likely due to the consumption of sequencing reagent, which is a common phenomenon in the Illumina high-throughput sequencing platform that based on sequencing by synthesis (SBS) technology [27, 28]. As expected, the first several bases have higher sequencing error rate than others. This could be due to the focusing of the sequencer’s fluorescence image sensor sensing element which may not be sensitive enough at the beginning of sequencing. As a result, the quality of acquired fluorescence reading is low. Recall that the sequences were appended with a pair of 20nt primer binding sites at both ends and hence the first several error-prone bases (around 6nt) have no influence on decoding, as the CRC test and RA encoding/decoding were designed by excluding the binding sites. In other words, a sequence will be identified as erased by the CRC decoder due to the errors in other positions (outside of primers).

In Fig. 3(C), a base content distribution of A, T, C and G along the reads is presented to show the distribution of the GC content. According to the principle of complementary bases, the content of AT and GC should be equal at each sequencing cycle and be constant and stable in the whole sequencing procedure. Notably, the observed mean GC content in a sequencing read and in each base position were both around 50% regardless of the first 20nt. The reason for the distribution in the first 20nt is due to the two binding sites in both ends. The distribution shows that the GC content of the sequenced oligos satisfies the biochemical constraint well and therefore ensures a stable sequencing process.

#### Data recovery analysis

To verify the code resilience of our designed RA error correction coding scheme, we studied the data recovery performance of the scheme over different coverages in Fig. 3(D). This gives us an estimate on the error resilience of the designed RA code against different dropout rates due to varied coverages. There exist some unusable raw sequences in the received sequencing reads because of their length being outside the acceptable range. To imitate different coverages (from 8x to 12x), we generated data sets of different sizes by performing random down-sampling on the usable raw sequences, in which the distribution of each message oligo may vary. For example, for coverage of 8x, we randomly down sampled the usable raw sequences to generate a data set of 96,000 raw sequences. For each coverage, we generated 5 different randomly down-sampled data sets and determined the average sequencing and decoding performance. For each raw sequence, we performed de-mapping to convert the nucleotide sequence to binary sequence and carried out CRC test to identify errorless/correct sequences. The average number of errorless sequences for each coverage is shown in Fig. 3(E) (black dots), as was expected, it increases with the increase of coverage. The errorless sequences were then fed to the RA decoder to recover the erroneous sequences. We observed that from coverage 10x and onwards, for each coverage, the decoder was able to recover the original sequences in 5 out of 5 random down-sampling experiments perfectly (green diamonds in Fig. 3(E)). This shows that the decoder is robust to recover erroneous data with the minimum coverage of 10x, where 3.3% of oligo sequences were in error (i.e., a dropout rate 3.3%)

## Discussion

As one of the recent emerging techniques in data storage field, the practical implementation of DNA-based data storage is challenged by the high cost and limited strand length due to the current DNA synthesis technology in synthetic biology. The limited manufacturing precision and techniques of DNA synthesis result in a low throughput and short length of valid DNA sequences, which leads to laborious work with high cost. While there has been great development in increasing the throughput of DNA synthesis, a coding scheme that enables error resilience towards biochemical mutations will greatly enhance data storing and retrieval against the error prone characteristic of the current or even future DNA synthesis and sequencing techniques. The scheme will allow the quality requirement of synthetic DNA production and sequencing to be relaxed, potentially reducing the cost. Furthermore, another instinctive approach to reduce the cost is to reduce the size of synthesized DNA required. This can be achieved through maximizing the amount of data stored per nucleotide or per oligo, which is realizable with a higher information density. With a coding scheme with a high information density, to store equivalent size of data, either the reduced DNA pool size or increased space for higher redundancy (that relax the manufacturing quality demand) could further reduce the cost of DNA synthesis, benefiting the practical implementation of DNA data storage. For instance, a reduction of 50% cost for storing per 1MB data (from ∼$4000 to ∼$2000 including sequencing and synthesis) could be found with an 1.80 bits/nt information density increase [15].

On the other hand, the retrieval of data from DNA storage systems relies on the redundant sequencing reads which is caused by the error-prone characteristic of current sequencing techniques. This sequencing redundancy leads to a limited number of distinct DNA sequences reading by a single sequencing kit. This results in high expense and resource cost for a large-sized sequencing request. Therefore, a storage scheme which enables effective error control code like the proposed is desired to reduce the demands on the sequencing redundancy, while promising full data recovery with lower sequencing coverage that reduces the cost.

Moving forward, We have introduced a DNA data storage with an optimized RA coding architecture and a hybrid mapping scheme, achieving competitive net information density and reliability against biochemical dropouts among existing works. The RA code is first harnessed as the error control code in DNA data storage context, overcoming the limitations of fountain code while presenting high error resilience. Meanwhile, comparing with the widely employed Reed-Solomon (RS) code in other studies, the RA code prevents from the local correlations in RS code that might hinder the practical utility for large-sized DNA data storage. From an error control coding aspect, we expect that the strategies based on DNA (quaternary) level balancing the trade-offs of coding complexity and efficiency might be the next step, despite of the concurrent challenge of meeting the biochemical constraints. The proposed hybrid mapping scheme is devised to comply with the biochemical constraints while offset the loss of mapping potential caused by the replacement of fountain code, ultimately attributing a very high net information density. The mapping scheme can be regarded as a constrained code that is subject to the biochemical constraints. The near-optimum mapping potential of the proposed infers that a further increment might not be readily found unless additional DNA alphabets (e.g., unnatural nucleotides [29], degenerate characters [15, 16]), which remains challenging in practice, are utilized. In addition, the system is optimally designed in terms of the realized capacity and retrieval reliability, subject to the current limitations in DNA data storage, such as the cost issue and length limit existing in the current DNA synthesis techniques, and inevasible biochemical bias and reading inaccuracy arisen from the biological processes and DNA sequencing techniques.

## Conclusions

To summarize, in this work, we have devised a high capacity DNA data storage with a reliable and optimized RA coding architecture and a novel hybrid mapping scheme, offering error resilience to the stored DNA molecules. We also show that the proposed scheme is highly competitive against existing schemes in terms of several performance aspects, among which the high mapping potential is demonstrated to be close to the upper theoretical boundary. We have experimentally tested the proposed scheme by encoding digital data into variable-length synthetic DNA oligos and subsequently decoded the data after sequencing the DNA molecules. The experiment shows results of the full recovery of digital data with low coverage of 10x, validating the proposed scheme. The theoretical analysis with the experiment shows that the proposed coding and mapping schemes enable a robust DNA storage system with a high capacity. With the proposed architecture and coding scheme, and together with the rapid development of synthetic biology technology, we expect a practical high-capacity DNA data storage system to be fast approaching in the future.

## Methods

### Encoded Data

We stored 379.1 KB source data into sets of oligos. Source files include five images and one text file. They are the images of Merlion (official mascot of Singapore), national flower of Singapore, national proclamation of Singapore, logo of Nanyang Technological University, Singapore (NTU), logo of National University of Singapore (NUS) and a text file containing Singapore national pledge in four languages (Fig. 1(B) and Additional file 1: Figure S1).

### Optimized repeat accumulate (RA) code

A systematic repeat-accumulate (RA) code was harnessed as the error control coding scheme due to its high error correction and low encoding/decoding complexity that grows linearly with the code length [19, 30] (see Additional file 1: Section S2). Besides, an optimization procedure (see Additional file 1: Section S2) was followed to design the RA code for DNA data storage.

### PCR amplification

The encoded DNA sequences appended with common binding sites 5’-ATACCCAAGGGTAAACAGCG-3’ and 5’-GCGGTTTCCAACCGGTAATA-3’ at the start and end were sent to TWIST Bioscience (TWIST Bioscience, US) where an oligo pool was synthesized. The synthesized oligo pool was then dissolved in 10 *μ*l nuclease free Tris-EDTA (TE) buffer, pH 8.0 (Sigma, US) to get oligo concentration of 37.9 ng/ *μ*l. PCR was carried out by using Q5®High-Fidelity DNA Polymerase (New England Biolabs, US). Primers 5’-ATACCCAAGGGTAAACAGCG-3’ and 5’-TATTACCGGTTGGAAACCGC-3’ obtained from Integrated DNA Technologies (IDT, US) were used for PCR of the oligo pools. 1 *μ*l oligo pool template, 2 *μ*l of each primer, 2 *μ*l dNTP, 1 *μ*l polymerase, 10 *μ*l reaction buffer and 32 *μ*l water were mixed for running 50 *μ*l PCR reaction. Instead of using routine thermocycling conditions of the thermocycler (Bio-Rad, US), the elongation time of 10 seconds and 9 cycles were used in this work. Nanodrop (Thermo Fisher Scientific, US) measurement and gel electrophoresis were performed to determine the PCR yield and existence of desired DNA band before sending the samples for sequencing.

### Sample preparation by Illumina Sequencing

Quality Control (QC) was performed by NovogeneAIT (NovogeneAIT, Singapore) before the PCR product was used for sequencing. QC was done by Agarose Gel Electrophoresis to check the existence of the right size band. Followed that, Qubit Fluorometer was used to determine the quantity of the PCR sample. DNA Library construction for Illumina Next-Generation Sequencing was then performed by using NEBNext Ultra II DNA Library Prep Kit (New England Biolabs, US). 500 pg −1*μ*g fragmented DNA was sheared in 50 *μ*g 1X TE (1 mM Tris-HCl, pH 8.0, 0.1 mM EDTA). This was followed by i) NEBNext end prep, to repair each DNA fragments by 5^′^ Phosphorylation and dA-Tailing; ii) Adaptor Ligation with NEBNext Adaptor; iii) Size selection of cleanup of adaptor-ligated DNA to remove uracil; iv) Enrichment of adaptor-ligated DNA by PCR amplification; v) PCR enrichment of adaptor-ligated DNA; and lastly vi) PCR reaction clean up. Samples were sequenced using HiSeq (Illumina) by NovogeneAIT.

## Supplementary information

**Additional file 1** High capacity DNA data storage with variable-length Oligonucleotides using Repeat Accumulate code and hybrid mapping: Supplementary information.

## Data Availability

The data that support the results of this study are available from the corresponding authors upon reasonable request.
