# Supplementary material for: High capacity DNA data storage with variable-length Oligonucleotides using repeat accumulate code and hybrid mapping
Source: J Biol Eng. 2019 Nov 21;13:89. doi: 10.1186/s13036-019-0211-2 (PMC6868767; doi:10.1186/s13036-019-0211-2)
Supplement: Supplementary file 1 — Additional file 1 High capacity DNA data storage with variable-length Oligonucleotides using Repeat Accumulate code and hybrid mapping: Supplementary information. [file 13036_2019_211_MOESM1_ESM.pdf]

High capacity DNA data storage with variable-length Oligonucleotides using Repeat Accumulate code and hybrid mapping: Supplementary information

Yixin Wang[1], Md. Noor-A-Rahim[4], Jingyun Zhang[2,3], Erry Gunawan[1], Yong Liang Guan[1], and Chueh Loo Poh[2,3]*

[1]School of Electrical & Electronic Engineering, Nanyang Technological University, 639798, Singapore, Singapore
[2]Department of Biomedical Engineering, National University of Singapore, 117583, Singapore, Singapore
[3]NUS Synthetic Biology for Clinical and Technological Innovation (SynCTI), Centre for Life Sciences, National University of Singapore, 117456, Singapore, Singapore
[4]School of Computer Science and IT, University College Cork, College Road, Cork T12 K8AF, Ireland

* To whom correspondence should be addressed.
Tel: +65 6516 7657; Email: poh.chuehloo@nus.edu.sg

S1. Detailed encoded source file and data structure

We encoded 379,050 bytes of source data with sizes ranging from 1.8 KB to 224.5 KB into 12,000 oligos, among which 11,400 oligos were for the user data and 600 oligos were for the redundancy that ensure error resilience. There are six source files including five images and one text file. Four images are the Merlion (the official mascot of Singapore)(https://en.wikipedia.org/wiki/Merlion), the national flower of Singapore(https://www.nhb.gov.sg/what-we-do/our-work/community-engagement/education/resources/national-symbols), the national proclamation of Singapore (https://mothership.sg/wp-content/uploads/2017/06/gpproclamation0908.jpg), the logo of Nanyang Technological University, Singapore (NTU), the logo of National University of Singapore (NUS) and the text file containing Singapore national pledge in four languages is also included (**Supplementary S1 Fig. 1**).

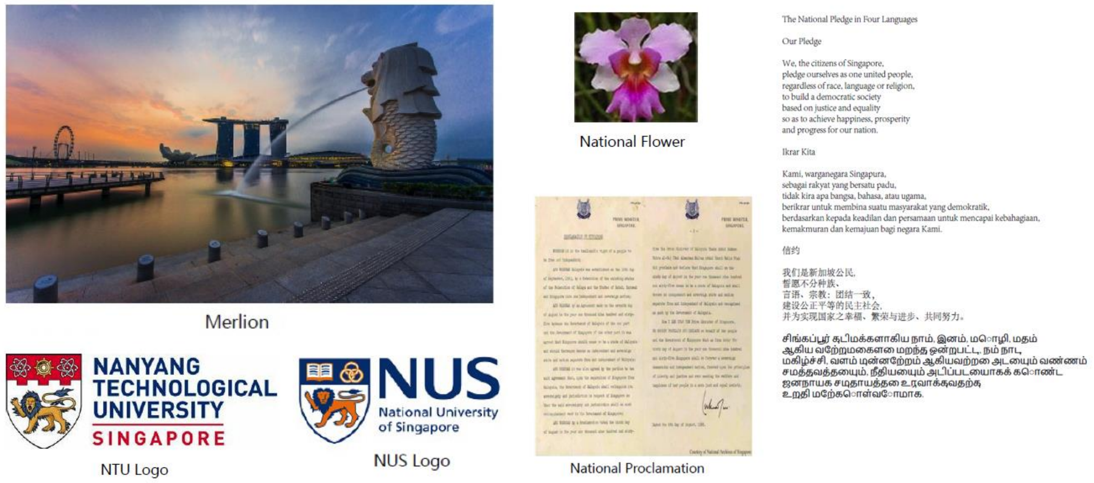

Supplementary S1 Fig. 1: Source files of the stored data. Five images and one text file are encoded from binary stream into 11,400 binary sequences and then transferred into 11,400 DNA oligos. Each file is encoded into different sizes of oligonucleotide (oligo) sets.

To ensure each encoded DNA oligo with the length under the limit of current synthesis technique (200nt), the length of encoded DNA sequences was set to be around 150nt after excluding 40nt for two 20nt primer sites. Considering the theoretical mapping potential (the number of bits encoded in one nucleotide) of 2 bits/nt, the length of binary sequence was thus set to be 300 bits. As we planned to store data in DNA oligos with size of 10^4 magnitude, we then set aside 14 bits from 300 bits for addressing distinct oligos ($14 > \log_2 10^4$). Moreover, we set aside another 20 bits for Cyclic Redundancy Check (CRC) for each 300 bits long sequence. Therefore, resulting in 266 bits of each sequence to encode user data. We segmented the user data into 11,400 binary sequences with each packet length of 266 bits. Then the repeat-accumulate (RA) code was applied to these user sequences and redundant/parity packets were generated. After that, each binary packet was appended with 14 bits as the address for ordering the position of each sequence in the whole user data set. Besides that, 20 bits was added CRC check for detecting the interior errors occurring within the sequence. Next, all the binary packets with a total length of 300 bits were mapped to DNA sequences according to the hybrid mapping strategy, resulting in DNA sequences with lengths ranging from 150nt to 159nt. Before sending the sequences for oligo synthesis, each DNA sequence was attached with with common binding sites 5'-ATACCCAAGGGTAAACAGCG-3' and

5'-GCGGTTTCCAACCGGTAATA-3' at the start and end. Finally, each synthesized oligo was in the length ranging from 190nt to 199nt. The structure of the data sequence and oligo sequence is shown in **Supplementary S1 Fig. 2**.

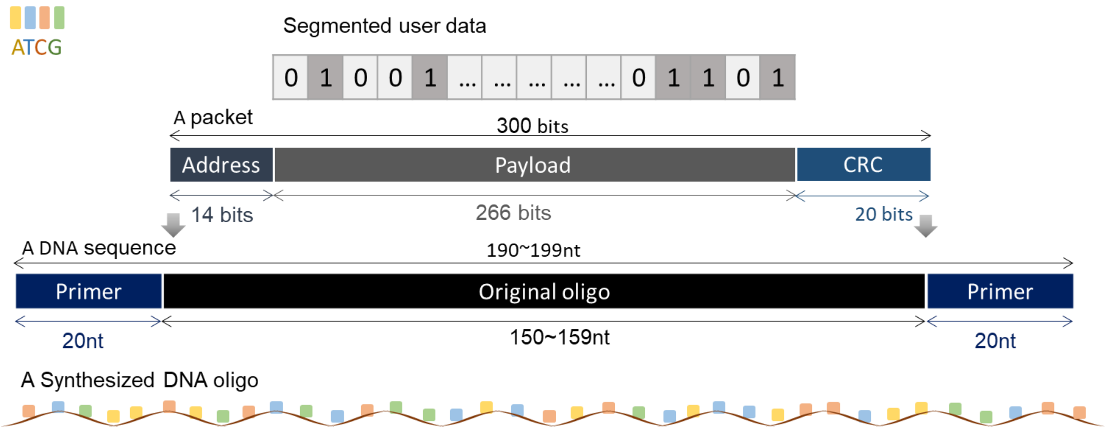

Supplementary S1 Fig. 2: The structure of a packet and a DNA sequence. Binary stream is segmented into packets with length of 266 bits and then each sequence is added with 20 bits for CRC check and 14 bits for addressing. The resulting 300 bits sequences are mapped to oligo sequences with a pair of 20nt primers attaching at both ends, resulting in the total lengths ranging from 190nt to 199nt.

S2. RA code design

In this work, we consider a systematic repeat accumulate (RA) code as error protection coding scheme. The rationale behind choosing RA code is their low encoding complexity. RA codes can be encoded by the serial concatenation of a repetition code, interleaver, combiner, and accumulator where the encoding complexity grows linearly with the code length (1,2). In general, a RA code can be represented by a bipartite graph, which consists of two types of variable nodes (namely information bit nodes and parity bit nodes) and one type of check nodes. The Tanner graph representation of a RA code is shown in **Supplementary S2 Fig. 3**, where the information bit nodes, check nodes, and parity bit nodes are correspondingly shown at the top, middle, and bottom of the graph. Each information bit node is connected to more than one check nodes. On the other hand, except the last parity bit node, each parity bit node is connected to exactly two check nodes. The parity bit nodes are connected to check nodes such that the ith parity bit node is connected to the ith and $i+1^{th}$check nodes. A RA code is

called (q, a) –regular code, if every information bit node is connected to exactly q check nodes and every check node is connected to exactly a information bit node. Thus, for (q, a) –regular RA code, the degrees of information bit nodes and check nodes become q and $a + 2$, respectively. In general, the degree distributions are characterized by the following functions (2):

- $v(x) = \sum_{i=2}^{q_{max}} v_i x^i$, where v_i is the fraction of information bit nodes of degree i.
- $h(x) = \sum_{i=2}^{a_{max}} h_i x^i$, where h_i is the fraction of check nodes of degree $i + 2$.
- $\lambda(x) = \sum_{i=2}^{q_{max}} \lambda_i x^{i-1}$, where λ_i is the fraction of edges that are connected to degree i information bit nodes.
- $\rho(x) = \sum_{i=2}^{a_{max}} \rho_i x^{i-1}$, where ρ_i is the fraction of edges that are connected to degree $i + 2$ check nodes.

Where q_{max} and a_{max} correspond to the maximum degree of variable nodes and check nodes, respectively. For the above mentioned degree distributions, the code rate becomes (2) :

$$R = \left(1 + \frac{\sum_j \rho_j / j}{\sum_j \lambda_j / j}\right)^{-1} \quad [1]$$

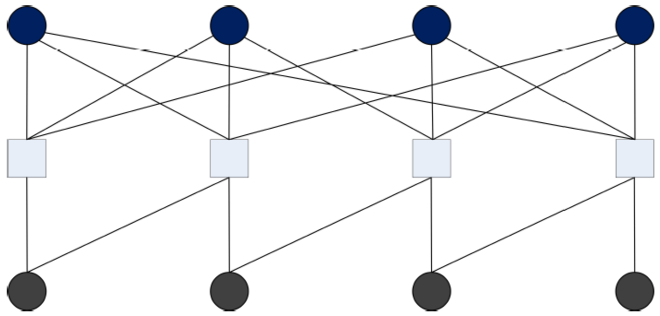

Supplementary S2 Fig. 3: Tanner graph representation of a (3,3)-regular RA codes. In the graph, the information bit nodes are represented by blue circle nodes, check nodes are represented by rectangular nodes, and parity bit nodes are represented by black circle nodes.

Instead of a conventional bit-level coding, we consider a packet-level coding. As described earlier, we segment a large digital file into smaller packets of the same size. We consider these packets as source packets which will be used to generate some redundant or parity packets. Note that every packet will be incorporated with CRC to detect errors in the packet. We will consider the packets that pass the CRC test as correctly recovered and others as erased.

Thus, the overall code design problem for the DNA storage becomes the code design for erasure channel. Let δ be the probability that error will occur in an oligo after the synthesis and sequencing processes. To ensure high reliability and robustness, in practice, code design is performed by considering slightly higher drop-out probability than the actual drop-out probability. Hence, we design the error protection code based on the dropout rate of oligos in synthesis and sequencing processes (3). We now show the density evolution analysis for RA code and then the optimization procedure to obtain capacity achieving RA code for a given δ.

A. Density Evolution

We present the density evolution of the RA code, which can be used to obtain the asymptotic threshold of a given RA ensemble. Let $x_s^{(l)}$ and $x_p^{(l)}$ be the erasure probabilities outgoing from the information nodes and the parity nodes, respectively to check nodes at iteration l. According to the density evolution analysis, we obtain the following update equations:

$$x_s^{(l)} = \delta\lambda\left\{1 - \left(1 - x_p^{(l-1)}\right)^2 \rho\left(1 - x_s^{(l-1)}\right)\right\}, \quad [2]$$

$$x_p^{(l)} = \delta\lambda\left\{1 - \left(1 - x_p^{(l-1)}\right)^2 h\left(1 - x_s^{(l-1)}\right)\right\}, \quad [3]$$

where $x_s^{(0)} = x_p^{(0)} = \delta$. From the above recursions, one can find the asymptotic threshold δ^* by the following expression,

$$\delta^* = max\left\{\delta\text{: } \lim_{l\to\infty} x_s^{(l)} = 0 \ \ and \ \ x_p^{(l)} = 0 \right\}.$$

We then optimize the RA code while utilizing the above density evolution equations $[2, 3]$.

B. Optimization

For simplicity, we first fix the check node degree distribution. Then we search for a variable node degree distribution $\lambda(z)$ such that, the resultant code becomes a capacity achieving for δ. With fixed $\rho(z)$, the rate of the RA code can be maximized by maximizing $\sum_j \lambda_j^{(r)}/j$. Thus, we obtain the following optimization problem.

$$\begin{cases} maximize & \sum_j \lambda_j/j \\ & x_s^{(l)} < x_s^{(l-1)}, \quad l = 1, 2, \ldots, l_{max} \\ & x_p^{(l)} < x_p^{(l-1)}, \quad l = 1, 2, \ldots, l_{max} \\ such\ \ that & \sum_j \lambda_j = 1 \end{cases} \qquad [4]$$

Finally, based on the dropout rate of oligos in synthesis and sequencing process that was used in (3), with the dropout rate of 1.3%, we designed the RA code such that the resultant code exhibited an asymptotic threshold higher than dropout probability 0.013. Following the optimization procedure described above, we design a RA code of rate 0.95, which gives an asymptotic threshold 0.0475. Thus, the resultant code shows only a gap of .0025 from the Shannon's capacity limit (0.05). The degree distribution pair obtained from the optimization procedure are $\lambda(x) = 0.277x + 0.132x^2 + 0.163x^3 + 0.055x^4 + 0.373x^9$ and $\rho(x) = x^{69}$. Due to the rate 0.95 code, we generated 600 redundant/parity packets and hence the total number of binary packets becomes 12,000. **Supplementary S2 Fig. 4** presents the error correction performance (in terms of oligo dropout probability after decoding) of the above-mentioned RA coding scheme.

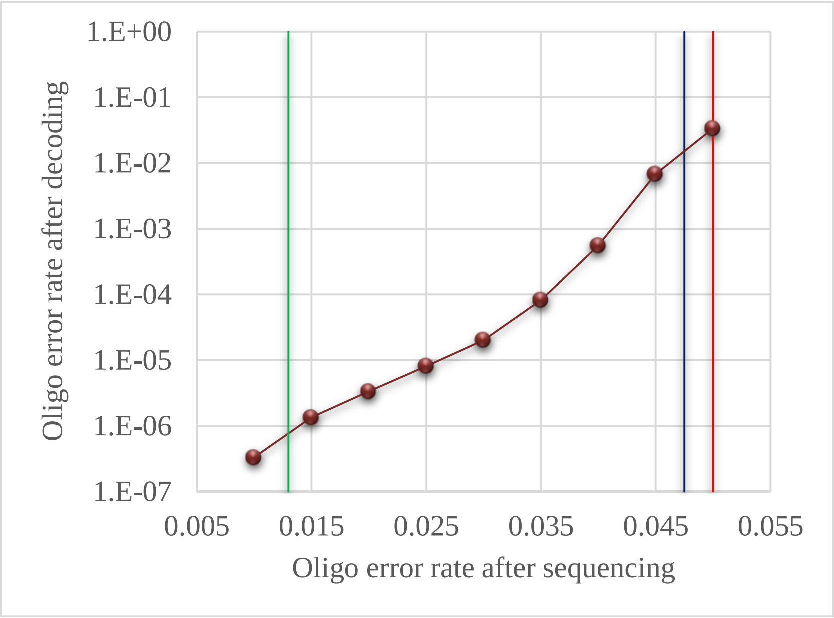

Supplementary S2 Fig. 4: Error performance of the proposed coding scheme. Here, red, blue and green vertical lines represent Shannon limit, asymptotic threshold, and drop rate of 0.013, respectively.

S3. Mapping potential and Information density

We compare the mapping potential and the information density of existing schemes reported in (3–9). The mapping potential (also known as coding potential) is defined as the number of bits encoded in one nucleotide and the net information density is defined as the number of user bits encoded in one nucleotide (3). The net information density can be calculated by the ratio of total bits of user data against total nucleotides of encoded DNA fragments. On the other hand, it can also be estimated by $\mathrm{NetInfo(bits/nt)} = L_i * R_c * R_m / L_p$, where R_c is the code rate of outer code, $\mathrm{L_i}$ is the length of information bits, $\mathrm{L_p}$ is the overall packet length, R_m is the mapping potential.

Church et al. (4) mapped 1 bit to 1 nucleotide (mapping potential 1 bit/nt) with 19 bits as the address for each encoding DNA block, and they did not use any error-correction code. This gave their scheme a net information density of $96\mathrm{bits}/115\mathrm{nt} = 0.83\ \mathrm{bits\ per\ nucleotide\ (bits/nt)}$.

Goldman et al. (5) used a ternary scheme with mapping potential 1.58 bits/nt and reported that their scheme achieved 0.0422 bytes per nucleotide after considering error-correction redundancy (fourfold repetition), addition of indexing and parity-check, yielding a net information density of 0.33 bits/nt.

Grass et al. (6) mapped two bytes to nine nucleotides with mapping potential 1.78 bits/nt and encoded 83 KB information into 4,991 DNA segments with a length of 158nt (including two adapters of total length of 41 for amplification and sequencing). The error-correction they used is Reed Solomon (RS) code. As the lengths of the primer sites are not counted in terms of net information density calculation, their scheme achieved a density of $83 * 1000 * 8/(4991 * 117) = 1.14\ \mathrm{bits/nt}$

Bornholt et al. (7) used the generated version of the coding scheme applied in Goldman et al.(5) with an improvement of 1.5x redundancy rather than 4x redundancy, and they encoded 151 KB of data into 45,652 DNA sequences of length 120 nucleotides (including two primer sites of total length of 40), yielding a density of $151 * 1000 * 8/(45652 * 80) = 0.33\ \mathrm{bits/nt.}$. However, they tested out their data on basic of Goldman's scheme. Therefore, the density they achieved was $0.33 * 4/1.5 = 0.88\ \mathrm{bits/nt}$ and the mapping potential is the same as

Goldman et al.(5).

Blawat et al. (8) mapped one byte to five nucleotides (with mapping potential 1.6 bits/nt) and stored 22MB of data in 900,000 DNA strands of length 190, yielding a density of $22 * 8 * 10^6/(9 * 10^5 * 190) \;=\; 1.03 \;\text{bits/nt}$

Erlich et al. (3) reported they achieved 1.98 bits/nt mapping potential and encoded 2,146,816 bytes of information using 72,000 oligos of length 152nt. The net information density is thus $2{,}146{,}816 * 8/(72000 * 152) \;=\; 1.57 \;\text{bits/nt}$.

Organic et al. (9) mapped every column of 160bits to DNA sequences of length 110 and 114 (with mapping potential of 1.45 bits/nt and 1.40 bits/nt). With RS code as the error correction code, they reported they have used 13,448,372 DNA sequences to encode 200.2 MB of data, yielding a net density of approximately 1.1 bits/nt.

Note that Yazdi et al. in (10,11) used different storage format for DNA storage where long double-stranded DNA blocks with a length of 1000 base were used. In (11), the scheme yielded a net information density of 1.74 bits/base due to this long DNA blocks format (1000 compared with 200). However, synthesizing long double-stranded DNA molecules is much expensive than synthesizing short single-stranded DNA molecules and longer DNAs are more prone to errors. Besides, it is worth to mention that our coding strategy does not rely on the length of synthesized DNA molecules. Thus, using this long DNA molecule storage unit, the high net information density performance will be maintained. For example, with an assumption of equivalent sequence length of the one used in Yazdi's work, and keeping the address length to be 14 bits and adding more bits for CRC redundancy (using 32 bits for CRC with a higher redundancy over 16 bits due to the increased length of encoded binary blocks), our coding scheme still has a higher net density of 1.84 bits/base over 1.74 bits/base. This is estimated by $\frac{\mathrm{L_i * Rc * Rm}}{\mathrm{L_p}} = \frac{(1000*1.98-14-32)*0.95*1.98}{1000*1.98} = 1.84 \;\text{bits/base}.$

For fair comparison reason, we exclude Yazdi's schemes from the comparison table as we consider the premise of a storage architecture based on the short single-stranded DNA fragments (oligos) pool that are more generally used.

To verify that the proposed coding scheme maintains high capacity performance with large data size, we assume to encode data of large sizes as some of the existing works *in silico*

(3,8,9). The number of bits used for indexing should satisfy $L_{id} \geq \log_2(N_{data}/R_c) - \log_2(L_P - L_{CRC} - L_{id})$, where L_{id} is the length of indexing needed for all encoded packets including user packets and redundancy packets with N_{data}/R_c bits in total. Note that for each magnitude, the packet length L_P is fixed to 300 to comply with the limitation of current synthetic biology (maximum ~160nt excluding primer binding sites), CRC length L_{CRC} is fixed to 20 to detect errors in 300bits packets. Thus, to encode 2MB of data as (3), we need 16 bits as indexing to indicate all encoded oligos. Therefore, we can obtain an estimated net information density of 1.66 bits/nt, calculated by $\frac{L_i * R_c * R_m}{L_p} = \frac{264*0.95*1.98}{300}$, according to the $\mathrm{NetInfo(bits/nt)} = L_i * R_c * R_m / L_p$. Similarly, we can calculate the density of 1.63 bits/nt and 1.61 bits/nt for storing 22MB (8) and 200MB (9) of user data with 20 bits and 23 bits as indexes, respectively.

Now we calculate the information density of our scheme. We encoded 266*11400 user bits into 12000 oligos with lengths ranging from 150 to 159. The information density is calculated by $\frac{11400*266}{(150*410+151*5429+152*4595+153*1032+154*339+155*123+156*51+157*16+158*4+159)} = 1.67$ bits/nt. Note that we use a 0.95 rate RS code and a hybrid mapping scheme with a mapping potential of 1.98 bits/nt. Together with the data structure we introduced in **Supplementary S1 Fig. 2**, with $L_i = 266, L_p = 300, R_c = 0.95, R_m = 1.98$, the estimate of the net information density be around 1.68 bits/nt, which is 7% higher than the last highest one reported in (3).

S4. Interleaved mapping

A. A pseudo code-based algorithm

A pseudo code-based algorithm for the proposed interleaved mapping is shown in **Supplementary Algorithm 1**. We have used following notations/functions in the algorithm:

- $SF\_mapping(.)$: Performs straightforward mapping on a binary sequence.
- $\mathrm{GC\_content}\ (.)$: Finds the ratio of GC content in a given DNA sequence.
- $\mathrm{int\_pattern}\ (i)$: i^{th} interleaving pattern.
- $\mathrm{Interleaving}(.,.)$: Performs interleaving on based straightforward mapped sequence with a given interleaving pattern.
- $HP\_run(\mathrm{F_{output}})$: Finds maximum length of homopolymer run in a given DNA sequence

Algorithm 1: Interleaved Mapping

Input : *binary_packet* which is a binary sequence containing CRC, address, and payload

1 /* Initialize the values of two variables ℓ and *flag* */

2 **Initialization:** $count = 0$, $flag = [A, T, G, C]$.

3 /* Perform straightforward mapping on the binary packet: $'00' \rightarrow A$, $'01' \rightarrow T$, $'10' \rightarrow G$, $'11' \rightarrow C$ */

4 $SF\_mapped = SF\_mapping(binary\_packet)$;

5 /* Find the GC content ration in SF_mapped */

6 $Ratio_{GC} = GC\_content\big(SF\_mapped\big)$

7 /* Check the GC content ratio constraint */

8 **if** $Ratio_{GC} > 60\%$ *or* $Ratio_{GC} < 40\%$ **then**

9   Exit the algorithm and perform variable-length constrained mapping

10 $\ell = \ell + 1$;

11 **if** $\ell = 5$ **then**

12   Exit the algorithm and perform variable-length constrained mapping

13 /* Perform interleaving on the mapped sequence with ℓ^{th} interleaving pattern. */

14 $Int\_output = Interleaving\Big(SF\_mapped, int\_pattern(\ell)\Big)$;

15 /* Add $\ell^t h$ element of *flag* at the end of Int_output */

16 $F\_output = Int\_output + flag(\ell)$

17 /* Find the maximum length of homopolymer run */

18 $L_{max} = HP\_run(F\_output)$

19 /* Check the Homopolymer run constraint */

20 **if** $L_{max} < 4$ **then**

21   F_output is a valid sequence for synthesis. Exit the algorithm.

22 **else**

23   Go to step 10.

Supplementary Algorithm 1

B. *An example of the interleaved mapping*

We now explain this algorithm using an example binary sequence '01111111110001010111'. The binary data is first mapped into DNA sequence 'TCCCCATTTC' according to the rule of mapping {00, 01, 10, 11} to {A, T, G, C}. We observe that although the resultant sequence satisfies the GC content, there are homopolymer runs over 3 nucleotides, i.e. '......CCCC......'. Thus, the sequence is then sent to the interleaver. A variable l with initial value of 0 is set to indicate the trial number of interleaving, which is also used to select the flag nucleotide for each output valid DNA sequence. One symbol from the set {A, T, G, C} corresponds to l in {1, 2, 3, 4}

will be appended at the end of the interleaved sequence, which acts as the flag to represent the interleaving pattern. We then check whether the resultant sequence meets the homopolymer run constraint. In this example, we assume the first iteration of interweaving is able to break the homopolymer run, so we set 'A' to indicate the decoder that the original DNA sequence is interleaved by the first pattern. However, in the case of failing to eliminate homopolymers within four trials, the original binary sequence will be sent to the second mapping method namely the variable-length constrained (VLC) mapping.

S5. Constrained code for the (4,0,2) run-length limit DNA storage system

A. Variable-length constrained sequence code

Constrained codes have been widely used as a collection of codes in the constrained systems, such as the optical memory systems and the digital communication systems, to satisfy specific channel constraints including the run-length limit, dc-free etc. (12). In (13), the variable-length constrained sequence code (VCSC) has been devised with high code rate and low complexity features that outperforms most block codes. Specifically, it reported that the code efficiency of VCSC approaches 99%, where the efficiency is the ratio of code rate to the capacity of the constraint and the capacity of the constraint is defined in [5] where $N(m)$ represents the number of constraint satisfying sequences of length m (14):

$$C_{CSC} = \lim_{m\to\infty} \frac{\log_2 N(m)}{m} \qquad [5]$$

In this work, we harness this code as a capacity-approaching mapping strategy for constructing sequences that satisfy the homopolymer constraint of DNA-based storage. The rationale behind VCSC is that it maps a set of variable-length source words to a set of variable-length codewords where both the source words and codewords are prefix-free to ensure the instantaneous encoding and decoding (13).

A general procedure to construct a capacity approaching VCSC can be summarized as follows (15): i) The complete or incomplete minimal sets are constructed based on the finite-state transition diagram (FSTD) of a specific constrained system where the elements and the concatenations of elements in the minimal set all satisfy constrains of the system. ii) Candidate codeword sets are constructed based on the full extension or partial extension of

the minimal set with a predefined depth or width, where extension means the concatenation of elements. iii) Determine the optimal mapping between the variable-length source words and the variable-length codewords of each potential candidate codeword set via geometric Huffman (NGH) technique and select the mapping that owns the highest average code rate. Note that with the binary i.i.d. sources, the average code rate only depends on the lengths of the source words and codewords, which is calculated by

$$\mathrm{R} = \frac{\sum_i 2^{-l_i} l_i}{\sum_i 2^{-l_i} o_i} \qquad [6]$$

where l_i and o_i represent the lengths of the ith pair of source word and codeword, respectively.

B. The (4,0,2) run-length limit (RLL) DNA storage system

The DNA-based storage can be considered as a constrained system with RLL because the maximum homopolymer run of this system is limited to 3 repetitive nucleotides. It can be denoted by $(\mathrm{M}, d+1, k+1)$ where $d+1$ and $k+1$ are the lower and upper boundaries of the allowable numbers of repetitive M-ary symbols (run-length). Specifically, this $(\mathrm{M}, d+1, k+1)$ RLL code can be achieved from a (M, d, k) constrained code via the change-of-state precoding, where d and k represent the minimum and maximum number of repetitive zero's between non-zero's, respectively. As the (M, d, k) constrained code can be directly constructed by FSTD, usually (M, d, k) is used to denote a $(M, d+1, k+1)$ RLL constrained system for convenience. Note that although the notation of M in $(M, d+1, k+1)$ and (M, d, k) are identical while they have different meaning. M in $(M, d+1, k+1)$ refers to the M-ary symbols in the system while M in (M, d, k) represents M different transition symbols that indicate the transitions among M-ary symbols in a $(M, d+1, k+1)$ RLL code. Hence, we can see the DNA-based storage as an (M, d, k) constrained system, where $M = 4$, as four different transition symbols are involved to indicate transitions among four different nucleotides, e.g., ‘0’ transits ‘A’ to ‘A’, ‘1’ transits ‘A’ to ‘T’, ‘2’ transits ‘A’ to ‘C’, and ‘A’ to ‘G’, $d = 0$ and $k = 2$ as there is no limit on the minimum homopolymer and the maximum homopolymer is 3 (which relates to $k + 1$).

The process of generating constraint-satisfying codewords for a (M, d, k) constrained

system can be modeled as the generation of Markov information source using FSTD where the transition of states is restricted by the system constraints, i.e., the states record the length of consecutively repetitive 0's, and any (M, d, k) constrained sequence (codeword) with arbitrary length can be generated by the labels of the edges of a path in the FSTD and subsequently transformed into the $(\mathrm{M}, d+1, k+1)$ RLL sequences via the precoder. The FSTD of has $k+1$ states and a $(k+1)*(k+1)$ adjacency matrix $\boldsymbol{D}(d_{ij})$, where each entry d_{ij} is the number of edges in the FSTD transiting state i to state j. The Markov model can also be used to derive the Shannon capacity of the constrained system using $C = \ log_2 \lambda_{max}$, where λ_{max} is the largest eigenvector of $\mathbf{D}$. Accordingly, we generate the FSTD of the homopolymer-constrained DNA data storage. Based on the generated FSTD, we can deduce the adjacency matrix $\mathbf{D}$ to evaluate the capacity of this constrained system,

where $\mathrm{D} = \begin{bmatrix} 3 & 1 & 0 \\ 3 & 0 & 1 \\ 3 & 0 & 0 \end{bmatrix}$

Therefore, the capacity of (4, 0, 2) homopolymer-constrained DNA storage is denoted as $C = \ log_2 \lambda_{max} = 1.982$.

Moving forward, from the FSTD, a minimal set can be found from enumerating all the words that originate from and end in a certain state. Based on the codeword set generating from the extension of the minimal set and the process of generating a Huffman coding tree, an optimal one-to-one assignment between the variable-length source words and the variable-length codewords is built. The rationale of the Huffman coding tree is to map the codeword with the short length to the source word with the high possibility of occurrence and verse vice. This will reduce the redundancy which is added due to the constraints. With the assumption of an approximately equal distribution of 0 and 1 in the source binary data, it is obvious that a source word with less composition size has a higher occurrence possibility. The possibility can be expressed by $\rho(s) = {\rho_0}^{l_0} * {\rho_1}^{l_1}$, where $l_0 + \ l_1 = l, \ l$ is the length of the source word, l_0, l_1 are the composition size of 0 and 1 and ρ_0, ρ_1 are the occurrence possibility of 0 and 1, respectively.

With the optimized mapping between source word and codeword, the mapping potential can be calculated by $\frac{2^{-2}*2*3+2^{-4}*4*3+2^{-6}*6*2+2^{-5}*5}{2^{-2}*3+2^{-4}*2*3+2^{-6}*3*2+2^{-5}*3} = 1.976 \ \ bits/nt$

S6. An example of variable-length constrained mapping

We use an example to help understand the VLC mapping strategy. Suppose that a binary user sequence is '1100-00-00-1101-01-111100-0', then we use the look-up table to encode the sequence into '01-1-1-02-2-001-1'. Notice that the last bit in the user data has no corresponding codeword, so we assign a codeword whose corresponding source word has the minimal suffix length with this bit as a prefix, here we map to '1' (alternative is mapping to '2'). It is worth to mention that the choice of the mapping of the last segment will not affect the decoding as the last bit de-mapped by any codeword will be the same. Then we pre-code this constrained code to RLL code with the change-of-state function $\mathrm{y}_j = y_{j-1} + x_j \pmod{\mathrm{M}}$, where y_j is the current output precoding symbol, y_{j-1} is the last output pre-coded symbol, x_j is the current input symbol, M is the alphabet size of the system(12). Note that the initial state is set to be 0. Following the function, we pre-code along the code and finally obtain '01-2-3-31-3-330-1'. At last, we convert the quaternary symbols to nucleotide symbols and thus get the DNA oligo sequence, denoted by 'AT-C-G-GT-G-GGA-T'.

S7. Additional information on the comparison table

We make the comparison of our scheme with other existing schemes that used the oligo pool storage format in terms of the following aspects in **Supplementary Table 2**:

- **Mapping potential:** the capacity of a nucleotide to encode binary bits.
- **Redundancy:** the ratio of the total synthesized oligos to oligos encoded user data.
- **Robustness to dropouts:** strategies used to resist dropouts, respectively.
- **Error correction/ detection:** ability to detect or correct errors that occurred in the processes of synthesis and sequencing.
- **Full recovery:** whether the decoder recovered all source data correctly.
- **Coverage:** the ratio of the number of reads used to full recovery to the number of encoded oligos
- **Net information density:** ratio of number of information bits to the number of synthesized nucleotides (excluding primers and adapter annealing sites for sequencing).
- **Realized capacity:** percentage of the net information density in the theoretical Shannon

capacity of the DNA storage system.

S8. Interleaved patterns

We use four interleaved patterns for the interleaved mapping, the first of which is a default pattern. Each interleaver is in the length of 150 as each DNA fragment mapped via the binary-to-quaternary mapping is in the length of 150 (300/2). Four nucleotides are used as the flag to indicate the interleaved pattern, they are ‘A’, ‘T’, ‘C’ and ‘G’. In the below table, the number in each cell of each pattern denotes the original position of the current nucleotide, i.e. the first nucleotide in pattern 2 is from the 123th nucleotide in original DNA fragment.

Supplementary Table 1: The four interleaved patterns which are tagged by four nucleotides as the flag

Pattern 1 (‘A’)						Pattern 2 (‘T’)					
1	26	51	76	101	126	123	115	86	144	37	71
2	27	52	77	102	127	99	11	103	104	64	23
3	28	53	78	103	128	32	101	38	93	31	141
4	29	54	79	104	129	40	54	100	117	49	126
5	30	55	80	105	130	22	105	58	8	27	137
6	31	56	81	106	131	34	30	76	67	110	65
7	32	57	82	107	132	92	133	143	84	61	2
8	33	58	83	108	133	91	149	81	139	88	132
9	34	59	84	109	134	146	45	89	90	50	114
10	35	60	85	110	135	119	77	42	97	87	4
11	36	61	86	111	136	112	60	28	138	26	18
12	37	62	87	112	137	127	113	128	83	43	85
13	38	63	88	113	138	35	147	145	59	124	75
14	39	64	89	114	139	6	150	17	106	94	24
15	40	65	90	115	140	121	74	131	79	19	95
16	41	66	91	116	141	55	78	41	142	102	39
17	42	67	92	117	142	148	72	120	5	44	13
18	43	68	93	118	143	3	62	47	48	130	9
19	44	69	94	119	144	96	70	111	108	15	66
20	45	70	95	120	145	136	129	98	53	73	20
21	46	71	96	121	146	68	107	80	29	1	57
22	47	72	97	122	147	16	134	14	21	125	122
23	48	73	98	123	148	140	51	46	25	36	10
24	49	74	99	124	149	135	33	56	52	116	12
25	50	75	100	125	150	69	7	63	109	82	118

Pattern 3 ('C')						Pattern 4 ('G')					
24	66	55	6	51	49	148	99	118	104	14	8
144	130	22	145	29	23	33	35	40	75	112	93
94	61	109	5	27	65	96	60	88	39	29	7
131	85	37	123	122	138	61	21	145	131	11	114
111	67	9	133	107	57	108	57	66	12	53	85
25	69	52	149	118	112	3	100	37	5	56	18
2	64	12	90	35	143	43	65	72	27	143	23
20	104	108	47	129	127	1	73	79	128	64	144
40	21	14	48	84	8	25	38	150	111	138	70
91	99	80	103	148	3	140	142	22	117	147	130
15	142	1	54	125	126	109	78	26	54	136	92
115	13	89	102	120	135	4	135	113	47	2	63
121	79	73	31	96	75	19	81	28	62	139	91
78	116	70	58	28	113	123	141	49	80	105	42
10	82	44	19	132	72	102	94	46	89	55	48
86	92	77	140	33	95	6	84	101	77	106	132
16	32	45	98	42	41	119	124	122	95	52	41
119	87	30	60	124	56	36	45	97	103	115	134
146	62	100	81	39	17	90	126	74	149	9	116
26	46	63	83	11	4	76	137	32	120	87	13
68	71	150	136	50	18	58	82	17	83	44	68
141	88	134	137	114	101	20	31	127	30	121	86
36	128	43	59	93	147	10	51	16	133	146	50
74	117	97	106	139	76	125	98	129	107	15	34
34	7	105	38	53	110	69	110	67	71	24	59

S9. Tables

Supplementary Table 2: The comparison with existing DNA data storage.

Scheme	Full recovery	Error correction/de tection	Robustness to dropouts	Coverage (x)	Redundancy	Mapping potential (bits/nt)	Net information density	Realized capacity
Church et al.	No	No	No	3000	1	1	0.83	45%
Goldman et al.	No	Yes	Repetition	51	4	1.58	0.33	18%
Grass et al.	Yes	Yes	RS	372	1	1.78	1.14	62%
Bornholt et al.	No	No	Repetition	40	1.5	1.58	0.88	48%
Blawat et al.	Yes	Yes	RS	160	1.13	1.6	1.03	50%
Erlich et al.	Yes	Yes	Fountain	10.5	1.07	1.98	1.57	86%
Qrganick et al.	Yes	Yes	RS	5	1.15	1.45	1.1	60%
This work	Yes	Yes	RA	10	1.05	1.98	1.67	91%

Supplementary Table 3: The detailed information of source data

Source file	File size (KB)	Number of Oligos
Merlion.jpg	224.5	6752
National flower.jpg	5.8	174
NTU logo.jpg	12.8	382
NUS logo.jpg	16.1	484
National proclamation.jpg	118.1	3552
National pledge.txt	1.8	55
Total	379.1	11400

Supplementary Table 4: The mapping look-up table based on VLC mapping

Source word	00	01	10	1100	1101	1110	111100	111101	1111
Codeword	1	2	3	01	02	03	001	002	003

Supplementary Table 5: The length of generated oligos via the heterogeneous mapping

Mapping scheme/Oligo length	150	151	152	153	154	155	156	157	158	159	Total
Interleaved mapping	0	5429	0	0	0	0	0	0	0	0	5429
VLC mapping	410	0	4595	1032	339	123	51	16	4	1	6571
Total	410	5429	4595	1032	339	123	51	16	4	1	12000

References

1. Divsalar D, Jin H, McEliece RJ. Coding theorems for turbo-like codes. In: Proc 36th Allerton Conf on Communication, Control and Computing. 1998. p. 201–10.
2. Jin H, Khandekar A, McEliece R, others. Irregular repeat-accumulate codes. In: Proc 2nd Int Symp Turbo codes and related topics. 2000. p. 1–8.
3. Erlich Y, Zielinski D. DNA Fountain enables a robust and efficient storage architecture. Science (80-). 2017;355(6328):950–4.
4. Church GM, Gao Y, Kosuri S. Next-generation digital information storage in DNA. Science (80-). 2012;1226355.
5. Goldman N, Bertone P, Chen S, Dessimoz C, LeProust EM, Sipos B, et al. Towards practical, high-capacity, low-maintenance information storage in synthesized DNA. Nature. 2013;494(7435):77–80.
6. Grass RN, Heckel R, Puddu M, Paunescu D, Stark WJ. Robust Chemical Preservation of Digital Information on DNA in Silica with Error-Correcting Codes. Angew Chemie Int Ed. 2015;54(8):2552–5.
7. Bornholt J, Lopez R, Carmean DM, Ceze L, Seelig G, Strauss K. A DNA-based archival storage system. ACM SIGOPS Oper Syst Rev. 2016;50(2):637–49.
8. Blawat M, Gaedke K, Huetter I, Chen X-M, Turczyk B, Inverso S, et al. Forward error correction for DNA data storage. Procedia Comput Sci. 2016;80:1011–22.
9. Organick L, Ang SD, Chen Y-J, Lopez R, Yekhanin S, Makarychev K, et al. Random access in large-scale DNA data storage. Nat Biotechnol. 2018;36(3):242.
10. Yazdi SMHT, Yuan Y, Ma J, Zhao H, Milenkovic O. A rewritable, random-access DNA-based storage system. Sci Rep. 2015;5:14138.
11. Yazdi SMHT, Gabrys R, Milenkovic O. Portable and error-free DNA-based data storage. Sci Rep. 2017;7(1):5011.
12. Immink KAS. Codes for mass data storage systems. Shannon Foundation Publisher; 2004.
13. Steadman A, Fair I. Variable-length constrained sequence codes. IEEE Commun Lett. 2013;17(1):139–42.
14. Shannon CE. A mathematical theory of communication. ACM SIGMOBILE Mob Comput Commun Rev. 2001;5(1):3–55.
15. Cao C, Fair I. Construction of minimal sets for capacity-approaching variable-length constrained sequence codes. In: Signals, Systems and Computers, 2016 50th Asilomar Conference on. 2016. p. 255–9.
